# Specific Dietary Components and Gut Microbiota Composition are Associated with Obesity in Children and Adolescents with Prader–Willi Syndrome

**DOI:** 10.3390/nu12041063

**Published:** 2020-04-11

**Authors:** Sonika Garcia-Ribera, Montse Amat-Bou, Eric Climent, Marina Llobet, Empar Chenoll, Raquel Corripio, Lourdes Ibáñez, Marta Ramon-Krauel, Carles Lerin

**Affiliations:** 1 Institut de Recerca Sant Joan de Déu, 08950 Barcelona, Spain; sonica94@hotmail.com (S.G.-R.); moamat@sjdhospitalbarcelona.org (M.A.-B.); mllobet@sjdhospitalbarcelona.org (M.L.); Libanez@sjdhospitalbarcelona.org (L.I.); mramonk@sjdhospitalbarcelona.org (M.R.-K.); 2 Endocrinology Department, Hospital Sant Joan de Déu, 08950 Barcelona, Spain; 3 ADM Lifesequencing, 46980 Valencia, Spain; eric.climent@adm.com (E.C.); Maria.Chenoll@adm.com (E.C.); 4 Service of Pediatric Endocrinology, Parc Taulí Hospital Universitari, Institut d’Investigació i Innovació Parc Taulí I3PT, Universitat Autònoma de Barcelona, 08208 Sabadell, Spain; rcorripio@tauli.cat; 5 CIBERDEM, Instituto de Salud Carlos III, 28029 Madrid, Spain

**Keywords:** Prader–Willi syndrome, childhood obesity, adiposity, dietary intake, gut microbiota

## Abstract

Prader–Willi syndrome is a rare genetic disorder associated with impaired body composition, hyperphagia, and excessive weight gain. Strict dietary restrictions from an early age is crucial to prevent or delay the early onset of obesity, which is the main driver of comorbidities in these patients. The aim of this study was to identify dietary and gut microbiota components closely linked to weight status of these patients. We studied a cohort of children and adolescents with genetic diagnosis of Prader–Willi syndrome (*N* = 31), in which we determined adiposity by Dual-energy X-ray absorptiometry (DXA) and dietary composition with 4-day food records. Furthermore, we obtained fecal samples to assess microbiota composition by 16S sequencing. Multivariate regression models showed that body mass index standard deviation score (BMI-SDS) and body fat mass were directly associated with saturated fat intake and meat consumption, and inversely associated with fruit consumption. Furthermore, the gut microbiome from normal weight patients was characterized by higher phylogenetic diversity compared to those overweight or obese, with differential abundance of several genera, including *Alistipes*, *Klebsiella*, and *Murimonas*. Notably, *Alistipes* abundance was inversely correlated to adiposity, lipid and glucose homeostasis parameters, and meat intake. Our results suggest that limiting meat and increasing fruit intake might be beneficial for body weight management in children and adolescents with Prader–Willi syndrome.

## 1. Introduction

Prader–Willi syndrome (PWS) is a rare genetic disorder that is caused by lack of expression of paternal inherited genes located in the chromosome 15q11-q13 region [1]. The disease is present in 1:10,000–20,000 individuals and its most frequent genetic mechanisms are deletions in the paternal chromosomal region (approximately 65%–75% of cases) followed by maternal disomy of chromosome 15 (approximately 20%–30% of cases); other less frequent causes include imprinting defects or translocations in that chromosomal region [1]. PWS is characterized by a wide range of developmental and behavioral disturbances, including hypogonadism and hormonal dysfunction, hyperphagia, hypotonia, altered body composition (higher fat and lower lean mass), short stature, as well as dysmorphic features [1]. A complex hypothalamic dysregulation is currently thought to be responsible for this phenotype.

Regardless of the genetic origin, pediatric patients show a very characteristic progression through different nutritional phases [2]. Feeding difficulties usually accompanied by failure to thrive appear during the first months of life. These are followed by a period of normal-rate weight gain until approximately two years of age. After this phase, weight gain starts steadily increasing even without a change in energy intake. At around four years of age, patients start showing increased appetite and interest in food, which precede the onset of hyperphagia [2]. Together with the insatiable hunger, aberrant behaviors typically occur in this latter phase, including food seeking behavior, constant thinking about food, withdrawn-depression symptoms, and social problems [3]. Therefore, these patients require close supervision by caregivers and strict food access control, with consequent negative effects on their quality of life and important distress for both patients and families.

The occurrence of both abnormal body composition (reduced lean mass) and insatiable hyperphagia leads to reduced energy expenditure and increased caloric intake, respectively, favoring energy accumulation and resulting in excess of adiposity [4]. In fact, PWS is the most common cause of genetic obesity. Obesity is the main driver of comorbidities in these patients, including respiratory difficulties, sleep apneas, hypertension, steatohepatitis, and type 2 diabetes, responsible for their premature mortality [5]. Thus, one of the main goals of therapeutic strategies is preventing or delaying the early onset of obesity in order to change its clinical progression. Current management strategies include early interventions involving physical and cognitive stimulation, growth hormone therapy, and early dietary recommendations. Based on their lower energy requirements, these recommendations include limiting caloric intake by 20%–40%, together with a well-balanced macronutrient distribution and high fiber intake [1,4,6,7,8]. However, despite noticeable improvements in care during the last decade, adequate weight control continues to be extremely challenging and patients often develop obesity and associated complications.

The gut microbiome has emerged as an important player in determining host health and energy balance [9,10]. Its composition is highly affected by diet [11,12], and several studies have shown a causative role of microbiota in developing obesity [10,13]. However, research on the gut microbiome in subjects with PWS is very scarce. To date, only two studies have compared the microbiome of subjects with PWS with obesity and controls also with obesity [8,14].

In this study, we aimed to identify dietary components closely linked to weight status and adiposity in children and adolescents with PWS. Furthermore, we analyzed the gut microbiome in these subjects to shed more light into the interrelationship between diet, gut microbiome, and obesity in this population.

## 2. Materials and Methods

### 2.1. Human Subjects

This study was approved by the Hospital’s ethical committee (code number PIC-73-17). Participants were recruited at the Hospital Sant Joan de Déu (Barcelona, Spain) and Parc Taulí Hospital Universitari (Sabadell, Spain), from September 2017 to June 2018. Both hospitals are reference centers for this disease in Catalonia. Inclusion criteria were genetic confirmation of PWS, age between 4.5 and 18-years-old, and absence of any major cognitive disorder that could preclude participation in the study. All parents signed the informed consent form; additionally, patients older than 12 years of age provided written informed assent. All participants except for one were on growth hormone therapy. Subjects had been patients at the hospital for at least one year before the study, and parents were proficient in nutrition and care related to PWS at the time of the study. Also, given that this disease is characterized by low muscle tone and patients are significantly more sedentary than the general population, we encourage enrollment in physical activity programs, which mostly includes individual sports (especially dance classes, swimming, and tennis).

### 2.2. Physiologic and Metabolic Variables

Fasting blood tests and anthropometric measurements were obtained at the study visit. A stool sample obtained up to 48 h before was also collected at this time. Weight and height were determined with a digital scale and stadiometer by a qualified nurse. Body composition was determined on a Prodigy Lunar^®^ DXA scanner (Lunar Corp., Madison, WI, USA). Age- and sex-adjusted body mass index standard deviation scores (BMI-SDS) were calculated using WHO AnthroPlus software (World Health Organization, Geneva, Switzerland) [15]. Blood biochemical measurements were performed at the hospital laboratory following standard procedures. Homeostatic model assessment of insulin resistance (HOMA-IR) was calculated as described previously [16]. Hyperphagia levels were assessed with the Hyperphagia Questionnaire for Clinical Trials (HQ-CT), a nine-question questionnaire (score 0-36) developed for patients with PWS.

### 2.3. Dietary Analysis

Parents filled a 4-day food diary (three weekdays and one weekend day) during the week prior to the study visit. Food records were discussed in a personal interview with the nutritionist during the study visit, where portion sizes and cooking methods were detailed. Participants were taking no probiotic or prebiotic supplements. Reviewed records were then analyzed with DIAL software [17], providing the macro- and micro-nutrient composition of the diet, as well as the average daily intake of different food groups. Considered food groups included grains, legumes, vegetables, fruit, meat, fish, dairy, eggs, oils and fat, and beverages (sodas and juices), and were calculated as % of total calorie intake. Energy intake was calculated as daily calorie intake and as % of daily recommended caloric intake (RCI) for each patient, based on age-, gender-, and BMI-matched subjects.

### 2.4. Gut Microbiota Analysis

DNA from stool samples was isolated following Yuan et al. [18] with minor modifications, with the aid of QIAamp PowerFecal DNA Kit (Qiagen, Hilden, Germany) to avoid bias in DNA purification toward misrepresentation of gram-positive bacteria. For massive sequencing, the hypervariable region V3-V4 of the bacterial 16s gene was amplified using key-tagged eubacterial primers [19] and was sequenced with a MiSeq Illumina Platform (Illumina, Sant Diego, CA, USA) following Illumina recommendations for library preparation and sequencing for metagenomics studies. An average of 85,000 reads per sample were obtained. The resulting sequences were split taking into account the barcode introduced during the PCR reaction, while R1 and R2 reads were overlapped using PEAR program version 0.9.125 providing a single FASTQ file for each of the samples. Quality control of the sequences was performed in different steps. First, quality filtering (minimum threshold of Q20) was performed using fastx tool kit version 0.013. Next, primer (16s rRNA primers) trimming and length selection (reads over 300nts) was done with cutadapt version 1.4.126. These FASTQ files were then converted to FASTA files, and UCHIME program version 7.0.1001 was used to remove chimeras that could arise during the amplification and sequencing step. Those clean FASTA files were BLAST27 against the National Center for Biotechnology Information (NCBI) 16s rRNA database using blastn version 2.2.29+. The resulting XML files were processed using a python script developed by ADM Lifesequencing (Valencia, Spain) to annotate each sequence at different phylogenetic levels.

### 2.5. Statistical Analysis

Data are shown as mean and standard deviation (SD) or *n* and percentage (%) for continuous and categorical variables, respectively. Student’s *t*-test or Chi-square test for continuous or categorical variables were used for group comparisons. Multivariate linear least-squares regression was applied to assess associations between % fat mass or BMI-SDS (dependent variables) and dietary variables (independent variables), adjusted by sex, age, physical activity, genotype, metformin use, and energy intake. Results are shown as effect size (B) with 95% confidence intervals (CI). For microbiome analysis, DESeq2 package from R (R Core Team, 2012) was used to generate a generalized linear model with fixed effects with negative binomial family to compare operational taxonomic unit (OTU) counts between groups, using group (normal weight (NW) and overweight or obesity (OWO)) as a fixed effect. Count normalization was made by rarefaction and significance assessed with the Wald test and the Benjamini–Hochberg correction to adjust for false discovery rate (FDR). Spearman correlation was used to assess associations of physiologic and dietary variables with the selected genus. A *p* < 0.05 was considered significant. Analyses were performed in JMP^®^ v14.3 (SAS, Cary, NC, USA).

## 3. Results

### 3.1. Subject Characteristics

Demographic and metabolic characteristics of participants are described in Table 1. Our cohort included 31 subjects (19 female and 12 male) within a range of ages (5 to 18 years-old) and BMI-SDS (−1.24 to 4.5 standard deviations). Subjects were divided into normal weight (NW, *n* = 12) and overweight or obesity (OWO, *n* = 19) groups based on BMI-SDS below or over 1 standard deviation, respectively, following the WHO criteria for overweight and obesity. Groups showed no differences in age, genotype distribution, or hyperphagia levels; a slightly higher proportion of female subjects and patients with metformin therapy was included in the OWO group (*p* = 0.075 and *p* = 0.061, respectively). Fifty-five per cent of subjects in our cohort performed more than 2 h per week of exercise, and there were no differences between NW and OWO groups. As expected, patients from the OWO group had significantly higher BMI-SDS and body fat mass than NW patients (Table 1). Although in general subjects showed a normal lipid profile (triglycerides <150 mg/dL, low-density lipoprotein (LDL)-cholesterol <130 mg/dL) [20], patients in the OWO group had higher LDL-cholesterol (*p* = 0.036) and a trend towards increased total cholesterol (*p* = 0.069) compared to the NW group (Table 1). Fasting glucose and HbA1c were also within the normal range in both groups (glucose <100 mg/dL, HbA1c <6%), while insulin levels were on the higher end of the normal range for our laboratory reference values. When comparing both groups, fasting insulin levels and HOMA-IR were two-fold higher in OWO than in NW group (*p* < 0.05 for both; Table 1), indicating some degree of insulin resistance in children and adolescents with overweight and obesity compared to normal weight patients.

### 3.2. Dietary Analysis of Children and Adolescents with Prader–Willi Syndrome

The dietary analysis revealed that energy intake was 80% the recommended caloric intake (RCI), in agreement with the recommendations for PWS (Table 2). Macronutrient distribution analysis showed a well-balanced diet within the recommended ranges [21]. Interestingly, we observed no differences in energy intake between OWO and NW groups (Table 2). Similarly, there were no major differences in macronutrient distribution or fiber intake, although diet from OWO subjects was slightly higher in fat and lower in carbohydrates than the diet from NW patients (Table 2). Regarding micronutrients, while intake of iron, folic acid, and vitamins A, B6, B12, and E achieved the recommended intake level, calcium and vitamin D were significantly below the threshold (Figure S1a). No differences in micronutrient intake were observed between NW and OWO groups (Figure S1b).

Regarding intake of the different food groups, normal weight patients showed significantly higher fruit and lower meat intake than those overweight or obese (Table 3). Intake of other food groups, including grains, legumes, vegetables, dairy, fish, eggs, oils and fats, and beverages were similar between groups.

### 3.3. Associations between Dietary Variables and the Degree of Obesity

To identify dietary determinants of the degree of obesity in these patients, we applied multivariate least-squares linear regression to assess potential associations between the different dietary variables and BMI-SDS or % of body fat mass. We observed no association between total calorie intake and BMI-SDS or adiposity (Table 4). Among macronutrients, only saturated fat intake was significantly associated with both variables (Table 4). Although markedly weaker, carbohydrate intake showed an inverse association with BMI-SDS (Table 4).

Regarding food groups, intake of grain, legume, vegetable, dairy, fish, eggs, oils and fats, or beverages did not correlate to BMI-SDS or fat mass (Table 5). Notably, meat intake showed a direct association with both BMI-SDS (*p* = 0.001) and body fat mass (*p* = 0.026) in these subjects, while fruit intake was inversely associated with both variables (Table 5). In agreement with the nutritional composition of these two food groups, meat consumption was correlated to saturated fat intake (Pearson coefficient *r* = 0.41, *p* = 0.022) and fruit consumption was correlated to carbohydrate intake (Pearson coefficient *r* = 0.43, *p* = 0.017).

### 3.4. Gut Microbiota Composition in Children and Adolescents with Prader–Willi Syndrome

**Figure 1:**
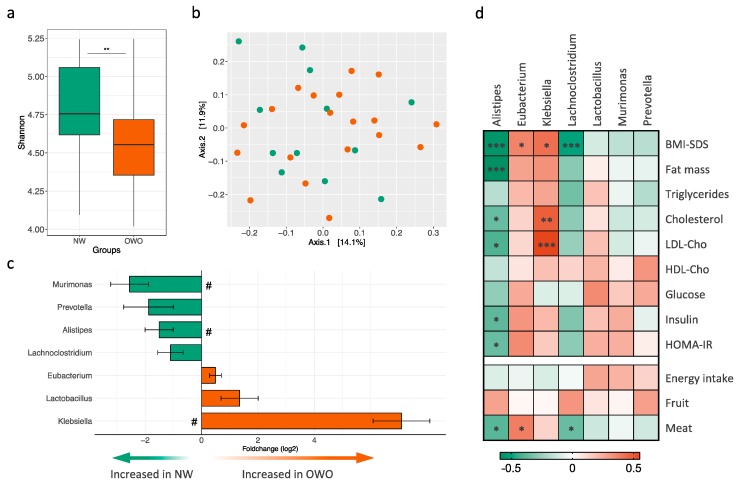
Gut microbiota analysis in children and adolescents with Prader–Willi syndrome. (**a**) Shannon index indicating α-diversity; significance between normal weight (NW; *n* = 12; green bars) and overweight and obesity (OWO; *n* = 19; orange bars) groups was assessed with Wilcoxon rank-sum test. (**b**) Principal coordinate analysis (PCoA) of unweighted UniFrac distances from all subjects (NW depicted as green circles, OWO as orange circles). (**c**) Fold change for genus with significantly different abundance in NW and OWO patients after Wald test; # indicates Benjamini–Hochberg adjusted *p* < 0.05. **d**) Heatmap of Spearman correlation coefficients between genera abundance and physiologic and nutritional variables. *, *p* < 0.05; **, *p* < 0.01; ***, *p* < 0.005. BMI-SDS, body mass index standard deviation score; LDL-Cho, low-density lipoprotein-cholesterol; HDL-Cho, high-density lipoprotein-cholesterol; HOMA-IR, homeostatic model assessment of insulin resistance.

The analysis of the gut microbiota by 16S profiling showed higher phylogenetic diversity in normal weight patients compared to those overweight or obese (Shannon index; Figure 1a). While there were no major changes in community structure as shown by permutational analysis of variance (PERMANOVA *p* = 0.146, Figure 1b), we observed differential abundance of seven microbial taxa at the genus level in the categorical comparison between NW and OWO groups (Figure 1c). Three genera had higher abundance in OWO compared to NW group, namely *Klebsiella*, *Lactobacillus,* and *Eubacterium*, while genera *Lachnoclostridium*, *Murimonas*, *Alistipes,* and *Prevotella* had lower abundance (Figure 1c). Three of these genera (*Klebsiella*, *Murimonas*, and *Alistipes*) were still significant after correction for multiple comparisons (FDR adjusted *p* < 0.05). OTU abundance at genus level and fold change between groups are listed in Table S1. Interestingly, we observed a gradation effect when further subdividing OWO patients into overweight (1 ≤ BMI-SDS < 2) and obesity (BMI-SDS ≥ 2) in both phylogenetic diversity and abundance of the selected genera (Figure S2). We next assessed potential correlations between the selected genera and physiologic variables. Correlation coefficients and significance levels are depicted in Table S2. Among them, lower *Alistipes* abundance was highly associated with higher body fat mass (Figure 1d). Furthermore, *Alistipes* was also inversely correlated to total and LDL-cholesterol levels, as well as glucose homeostasis parameters including fasting insulin levels and HOMA-IR (Figure 1d). Among the other genera, only *Klebsiella* showed a correlation to total and LDL-cholesterol (Figure 1d). Notably, while fruit intake was not associated to abundance of any of these genera, meat consumption was directly correlated to *Eubacterium* and inversely correlated to *Alistipes* and *Lachnoclostridium* abundance.

## 4. Discussion

Dietary management is one of the critical aspects that determine health and quality of life in patients with PWS. Based on their lower energy requirements, current dietary recommendations for PWS are based on a 20%–40% caloric restriction [1,4,6]. Different types of calorie-restricted diets have been proposed to improve weight management in patients with PWS [2,6,8,22]. In addition to energy restrictionS, a well-balanced macronutrient distribution and high fiber content are also important for successful weight control [7,8]. In agreement with the general recommendations, children and adolescents from our cohort had an average of 20% calorie restriction in their diets, with a well-balanced macronutrient distribution and a fairly high fiber intake. These patients also had an adequate intake of micronutrients, except for calcium and vitamin D which were below the recommended intakes. Due to the calorie restriction, dietary deficits in some micronutrients often occur in patients with PWS [23,24]. Interestingly, both calcium and vitamin D were also found to be below the recommended intake in a US-based cohort of youth with PWS [24].

Our results showed no differences in hyperphagia scores or energy intake between normal weight subjects and those overweight or obese, indicating that total calories were not contributing to obesity in this population. These data are in agreement with a study by Miller et al. showing that a group of children with PWS that followed a well-balanced macronutrient distribution diet achieved much better weight control than another group that followed a simple energy-restricted diet, even when both diets had similar caloric content [7]. All together, these data strongly suggest that diet quality plays an important role in weight management of patients with PWS. Indeed, our results show that those patients with lower meat and higher fruit consumption showed better weight management independently of total calorie intake.

From observational investigations to longitudinal cohorts and intervention studies, a myriad of studies has analyzed the dietary determinants of obesity in the general population. However, due to the complex etiology of obesity with multiple genetic and environmental factors and confounders, research has only been able to identify very few nutrients associated with weight management, including fiber, fat, fruit and vegetable intake, sugar-rich drinks, or energy-dense micronutrient-poor foods [25,26,27]. It is worth mentioning that the parents of children with PWS are generally extremely involved in their care and nutrition plan, as shown by the well-balanced macronutrient distribution in their diets. Furthermore, sugary drinks or high-energy foods are practically absent from their diets, and most likely do not significantly contribute to weight gain in these children.

While fat intake was once considered an important dietary determinant of obesity, a number of studies have now shown only weak or no association of total fat intake with weight gain [28,29,30]. In the Nurses’ Health Study, higher intake of saturated fat from animal origin showed a much stronger association to weight gain than total fat intake [30]. Consistent with these data, we observed that saturated fat intake was associated with both BMI-SDS and adiposity in our cohort. Saturated fat and meat intake were correlated to each other in our cohort, indicating that meat was an important source of saturated fat from animal origin. These data suggest that meat consumption might favor energy accumulation in the context of PWS, in agreement with other studies that have linked meat consumption, especially red and processed meat, with BMI in adult general population [31,32].

More consistent in the literature is the link between fiber intake and weight status [25], and increasing dietary fiber content is a general recommendation for patients with PWS. In fact, higher dietary fiber intake is associated with better weight management and weight loss in children and adolescents with PWS [7,8]. In our study, we observed no association of fiber intake with BMI-SDS or adiposity. This could be explained by the relatively high fiber content that was already present in their diet. Increasing fruit and vegetable intake has also been favor as a strategy for weight loss [26]. While we observed no association of obesity with vegetable intake in our cohort, lower fruit intake was correlated to higher BMI-SDS and adiposity.

During the last decade, an increasingly large number of studies have implicated the gut microbiome in host energy homeostasis and body weight management [10,13,33]. However, only two studies have examined the gut microbiome in subjects with PWS, both comparing the microbiota from patients with PWS and obesity to controls with obesity [8,14]. A study in children with obesity found no major differences in microbiota diversity and composition between patients with PWS and controls [8]. Conversely, a recent study in adult subjects with obesity observed higher phylogenetic diversity and differential abundance of certain taxa in subjects with PWS compared to matched controls [14]. Several factors could explain this apparent discrepancy between the studies, including different age-ranges (children versus adults), geographical localization, and ethnicity. Furthermore, in the adult study no differences in microbiota composition between subjects with PWS and their non-PWS parents were observed [14], suggesting that diet and other environmental factors could be playing an important role in determining gut microbiome structure. In our study, we compared the microbiome from normal weight patients and overweight or obese patients in the context of PWS. Notably, we observed lower phylogenetic diversity in overweight and obese patients compared to patients with normal weight. Indeed, decreased microbiota richness has been consistently associated with obesity and poor metabolic health in the general population, including impaired glucose homeostasis and increased adiposity [33,34].

Our study has identified several microbial taxa at the genus level associated with obesity status in children and adolescents with PWS. Three of these genera sustained adjustment for multiple comparisons, namely *Klebsiella*, *Murimonas*, and *Alistipes*. Among these genera, *Alistipes* showed a highly significant inverse association with body fat mass and was also inversely correlated to cholesterol levels and insulin resistance in our cohort. These data indicate that lower *Alistipes* abundance is linked to a poor metabolic health phenotype, at least in the PWS context. Interestingly, other studies have found lower *Alistipes* abundance in individuals with obesity compared to normal weight controls [35,36,37,38]. Furthermore, *Alistipes* was identified as a predictor of successful weight loss in a two-year intervention in adult subjects with obesity [39]. Together with these data, our results support a potential beneficial role of *Alistipes* in the context of obesity and metabolic health.

Our results showed that lower *Alistipes* abundance was also linked to higher meat intake. In fact, diet is one of the main determinants of gut microbiota composition [11,12]. In patients with PWS, Zhang et al. showed that a 30-day intervention with a diet rich in non-digestible carbohydrates led to changes in the gut microbiome and improved body weight and metabolic health in children with PWS [8]. By means of microbiota transplants into gnotobiotic mice, the authors demonstrated that these changes in the microbiome contributed to the beneficial effects of the intervention [8]. Thus, modulating the gut microbiome with probiotic supplementation or specific dietary patterns could provide a therapeutic strategy for PWS. Further research is warranted to determine whether meat consumption modulates intestinal abundance of *Alistipes* or other microbial taxa impacting obesity status and metabolic health.

The limitations of this study include that the dietary analysis is based on parental reported food diaries; while in general the parents and caregivers are well-trained regarding the quality of the diet and are proficient in filling up food diaries, we cannot exclude potential omissions due to foods consumed without parental knowledge. Furthermore, all subjects are from a specific geographical area (Catalonia), and further studies would be necessary to determine whether meat and fruit intake are also associated with the degree of obesity in patients with PWS from other regions of the world, with different dietary and lifestyle patterns. Finally, this is a cross-sectional study that describes associations between dietary variables, gut microbial abundances, and obesity; therefore, causality cannot be inferred.

## 5. Conclusions

In summary, our results indicate that obesity status and adiposity in children and adolescents with PWS are not associated with total caloric intake but correlate to higher meat and lower fruit consumption. Further studies are warranted to determine whether limiting meat and increasing fruit consumption can provide a strategy for weight control in children with PWS.

## Figures and Tables

**Table 1:** Demographic and metabolic characteristics of participants.

Variables	All Subjects (*N* = 31)	NW (*n* = 12)	OWO (*n* = 19)	*p*-Value ^a^
Gender (Females)	19 (61%)	5 (42%)	14 (74%)	0.075
Age (years)	12.0 (4.0)	11.4 (3.9)	12.4 (4.2)	0.498
Pre-pubertal status	12 (39%)	6 (50%)	6 (32%)	0.306
Genotype (Deletions)	18 (58%)	5 (42%)	13 (68%)	0.243
Hyperphagia (HQ-CT Score)	6.8 (6.0)	7.3 (7.4)	6.6 (5.1)	0.787
Physical activity (>2 h/week)	17 (55%)	8 (67%)	9 (47%)	0.290
Growth hormone therapy	30 (97%)	12 (100%)	18 (95%)	0.317
Metformin therapy	8 (26%)	1 (8%)	7 (37%)	0.061
BMI-SDS	1.51 (1.38)	0.22 (0.55)	2.32 (1.09)	**<0.001**
Body fat mass (%)	43.2 (8.4)	35.9 (3.9)	47.9 (7.0)	**<0.001**
Lipid profile				
Triglycerides (mg/dL)	70 (25)	64 (26)	74 (25)	0.294
Cholesterol (mg/dL)	170 (36)	155 (34)	179 (35)	0.069
LDL-cholesterol (mg/dL)	102 (31)	88 (30)	112 (28)	**0.036**
HDL-cholesterol (mg/dL)	56 (13)	55 (13)	58 (14)	0.529
Glucose metabolism				
Glucose (mg/dL)	87 (9)	85 (10)	88 (9)	0.352
HbA1c (%)	5.3 (0.2)	5.2 (0.3)	5.3 (0.2)	0.692
Insulin (mU/L)	12.6 (9.2)	8.0 (7.2)	15.6 (9.3)	**0.017**
HOMA-IR	2.82 (2.18)	1.76 (1.62)	3.49 (2.26)	**0.020**

Data are shown as mean and standard deviation (SD) for continuous variables and *n* and percentage (%) for categorical variables. ^a^, group differences were assessed with Student’s *t*-test (continuous variables) or Chi-square test (categorical variables). Bold font represents *p* < 0.05. HQ-CT, hyperphagia questionnaire for clinical trials; BMI-SDS, body mass index standard deviation score; LDL, low-density lipoprotein; HDL, high-density lipoprotein; HOMA-IR, homeostatic model assessment of insulin resistance.

**Table 2:** Dietary intake analysis of participants.

Dietary Intake	All Subjects *N* = 31	NW *n* = 12	OWO *n* = 19	*p*-Value
Energy Intake (kcal/day)	1571 (349)	1525 (308)	1600 (378)	0.552
(% RCI)	80 (22)	84 (26)	78 (20)	0.493
Protein (% kcal)	17.7 (2.7)	17.3 (2.7)	18.0 (2.9)	0.530
Fat (% kcal)	32.7 (6.0)	31.1 (6.1)	33.7 (5.9)	0.256
SFA (% kcal)	9.4 (2.6)	8.6 (2.7)	9.9 (2.4)	0.170
MUFA (% kcal)	14.9 (2.7)	14.1 (2.8)	15.4 (2.6)	0.198
PUFA (% kcal)	5.1 (1.3)	5.2 (1.2)	5.0 (1.3)	0.727
Carbohydrate (% kcal)	46.6 (5.6)	48.2 (4.6)	45.5 (6.0)	0.176
Fiber (g/day)	23.8 (12.0)	24.9 (10.0)	23.1 (13.4)	0.681

MRI, macronutrient recommended intake; NW, normal weight; OWO, overweight and obesity; RCI, recommended caloric intake; SFA, saturated fatty acids; MUFA, monounsaturated fatty acids; PUFA, polyunsaturated fatty acids. Data are shown as mean and SD. Group differences were assessed with Student’s *t*-test.

**Table 3:** Food group intake analysis of participants in the study.

Food Groups	All Subjects *N* = 31	NW *n* = 12	OWO *n* = 19	*p*-Value
Grains	27.6 (7.0)	25.6 (8.2)	29.0 (6.1)	0.235
Legumes	4.4 (3.6)	4.4 (4.4)	4.3 (3.2)	0.960
Vegetables	5.4 (2.1)	6.0 (2.0)	5.1 (2.1)	0.245
Fruit	11.8 (6.5)	14.6 (5.9)	10.0 (6.3)	**0.050**
Dairy	16.9 (6.6)	18.4 (8.3)	16.0 (5.2)	0.394
Meat	9.9 (5.8)	7.0 (3.8)	11.7 (6.3)	**0.015**
Fish	3.6 (1.7)	3.9 (1.7)	3.5 (1.8)	0.499
Eggs	1.7 (1.2)	1.8 (1.1)	1.7 (1.2)	0.946
Oils and fats	11.6 (2.6)	11.9 (3.0)	11.4 (2.4)	0.589
Beverages	1.6 (2.0)	1.6 (1.9)	1.7 (1.6)	0.899

NW, normal weight; OWO, overweight and obesity. Food group data are shown as mean and SD of % of total calorie intake. Group differences were assessed with Student’s *t*-test. Bold font represents *p* < 0.05.

**Table 4:** Associations between dietary variables and adiposity or BMI-SDS.

Dietary Variables	BMI-SDS		Body Fat Mass (%)	
	B (CI 95%)	*p*-Value	B (CI 95%)	*p*-Value
Caloric intake (kcal/day) ^a^	0.00 (0.00 to 0.00)	0.293	−0.01 (−0.02 to 0.00)	0.133
Protein (% kcal)	0.11 (−0.11 to 0.33)	0.302	0.11 (−1.18 to 1.41)	0.856
Fat (% kcal)	0.08 (0.00 to 0.17)	0.061	0.44 (−0.06 to 0.94)	0.084
SFA (% kcal)	**0.26 (0.06 to 0.47)**	**0.014**	**1.49 (0.31 to 2.67)**	**0.016**
MUFA (% kcal)	0.16 (−0.02 to 0.35)	0.081	0.88 (−0.20 to 1.96)	0.105
PUFA (% kcal)	−0.12 (−0.56 to 0.32)	0.571	−1.09 (−3.61 to 1.43)	0.379
Carbohydrates (% kcal)	−**0.09 (**−**0.18 to** −**0.01)**	**0.** **038**	−0.39 (−0.92 to 0.14)	0.145
Fiber (g/day)	−0.03 (−0.08 to 0.02)	0.168	−0.21 (−0.50 to 0.08)	0.144

Linear regression models were adjusted by sex, age, physical activity level, genotype, metformin use, and caloric intake. ^a^, model not adjusted by caloric intake. B, effect size from the multivariate regression model; CI, confidence interval; SFA, saturated fatty acids; MUFA, monounsaturated fatty acids; PUFA, polyunsaturated fatty acids. Bold font indicates *p* < 0.05.

**Table 5:** Associations between food group intake and BMI-SDS or adiposity.

Food Groups	BMI-SDS		Body Fat Mass (%)	
	B (CI 95%)	*p*-Value	B (CI 95%)	*p*-Value
Grains	0.03 (−0.04 to 0.11)	0.360	0.19 (−0.25 to 0.63)	0.375
Legumes	−0.05 (−0.23 to 0.12)	0.535	−0.19 (−1.20 to 0.83)	0.707
Vegetables	−0.22 (−0.48 to 0.04)	0.098	−0.64 (−2.21 to 0.93)	0.406
Fruit	−**0.12 (**−**0.2 to** −**0.04)**	**0.008**	−**0.64 (**−**1.14 to** −**0.14)**	**0.015**
Dairy	−0.01 (−0.11 to 0.08)	0.751	−0.08 (−0.62 to 0.47)	0.776
Meat	**0.12 (0.05 to 0.20)**	**0.002**	**0.52 (0.05 to 0.99)**	**0.033**
Fish	−0.12 (−0.46 to 0.21)	0.459	−0.95 (−2.86 to 0.97)	0.318
Eggs	0.23 (−0.24 to 0.69)	0.329	1.45 (−1.24 to 4.15)	0.276
Oils and fat	−0.05 (−0.27 to 0.17)	0.632	−0.03 (−1.31 to 1.26)	0.966
Beverages	−0.17 (−0.44 to 0.11)	0.216	−0.38 (−2.01 to 1.25)	0.632

Model adjusted by sex, age, physical activity level, genotype, metformin use, and caloric intake. B, effect size from the multivariate regression model; CI, confidence interval. Bold font indicates *p* < 0.05.

## Supplementary Materials

The following are available online at https://www.mdpi.com/2072-6643/12/4/1063/s1, Figure S1: Micronutrient intake in children and adolescents with Prader–Willi syndrome, Figure S2: Microbiome composition in normal weight (NW), overweight (OW), and subjects with obesity (OB); Table S1: Differentially abundant operational taxonomic units (OTUs) between NW and OWO groups; Table S2: Correlations between selected taxa and the indicated physiologic and dietary variables.
