# Supplementary material for: Specific Dietary Components and Gut Microbiota Composition are Associated with Obesity in Children and Adolescents with Prader–Willi Syndrome
Source: Nutrients. 2020 Apr 11;12(4):1063. doi: 10.3390/nu12041063 (PMC7230364; doi:10.3390/nu12041063)
Supplement: Supplementary file 1 [file nutrients-12-01063-s001.zip › Supplementary Figure S1 and S2.pdf]

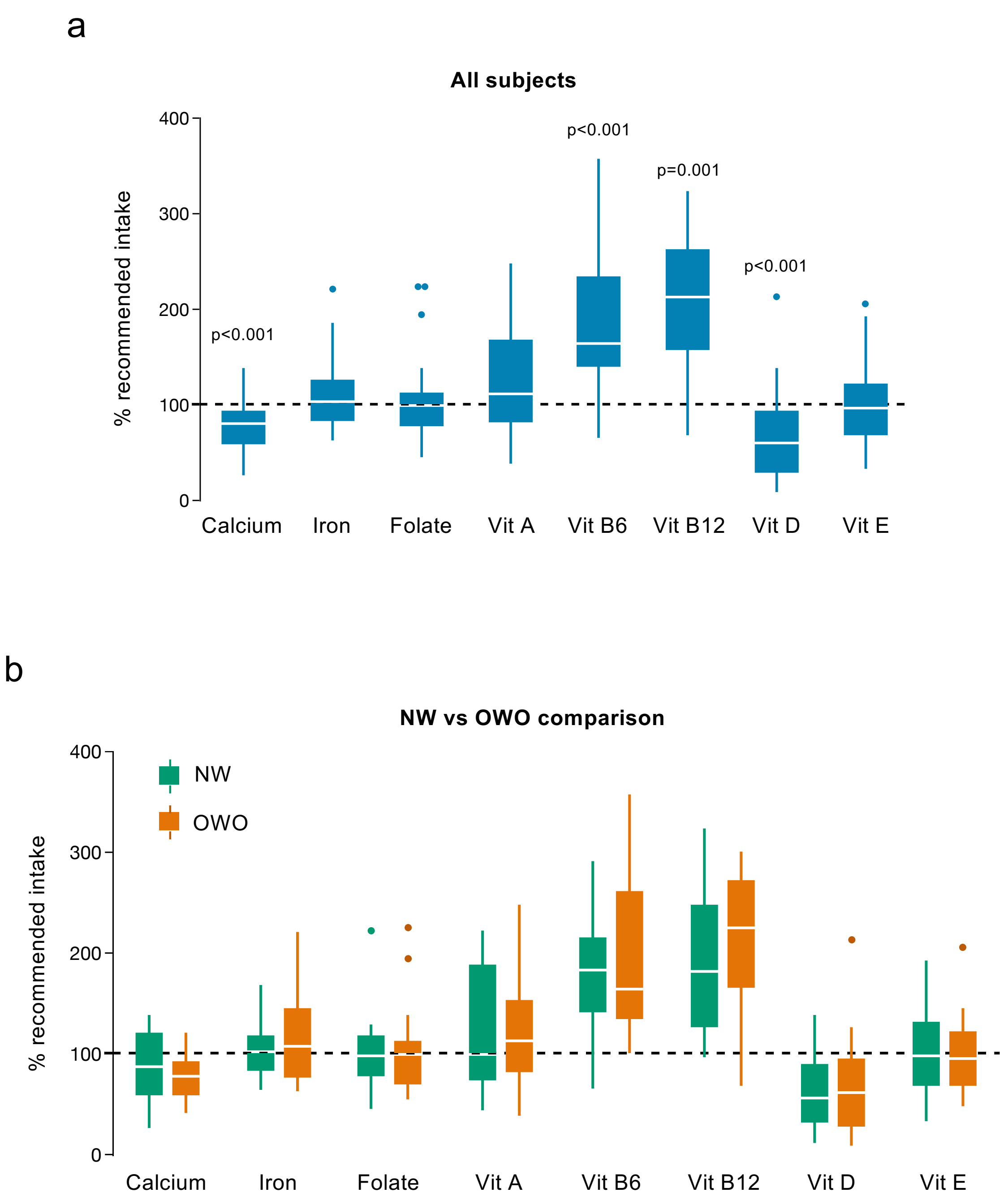

Figure S1. Micronutrient intake in children and adolescents with Prader-Willi syndrome. Micronutrient intake in children with PWS as a percent of daily recommended intake is shown. **a**) Data for all subjects is shown; significance for deviation from the 100% recommended intake for each micronutrient was assessed with one-sample *t*-test comparing the mean intake against a hypothetical mean (100%). **b**) Data for NW (n=12, green) and OWO (n=19, orange) groups are shown; no statistically significant differences were found between groups (assessed with Student's *t*-test). Box plots represent the median and interquartile ranges.

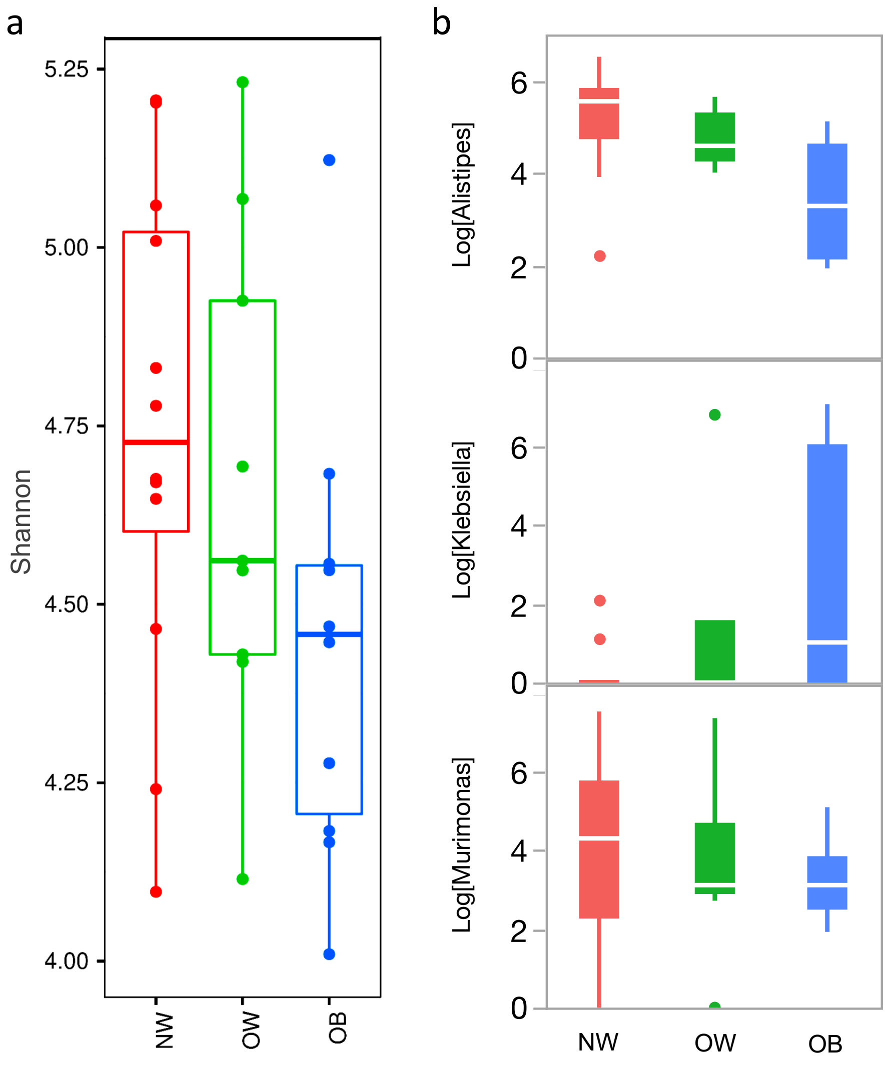

Figure S2. Microbiome in normal weight, overweight, and subjects with obesity.
a) Shannon index indicating α-diversity in normal weight (NW, n=12, red bars), overweight (OW, n=9, green bars), and subjects with obesity (OB, n=10, bleu bars). b) Abundance of Alistipes, Klebsiella, and Murimonas, expressed as log-transformed number of counts.

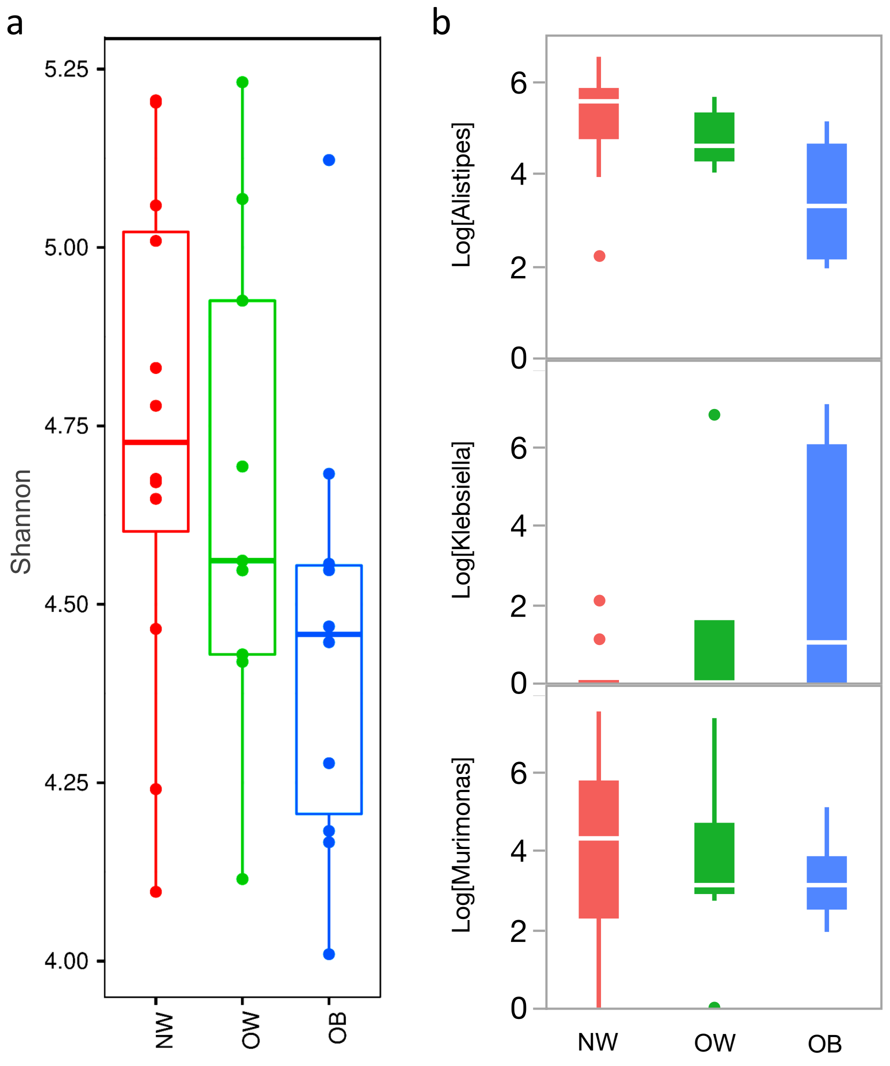

Figure S2. Microbiome in normal weight, overweight, and subjects with obesity. a) Shannon index indicating α-diversity in normal weight (NW, n=12, red bars), overweight (OW, n=9, green bars), and subjects with obesity (OB, n=10, bleu bars). b) Abundance of Alistipes, Klebsiella, and Murimonas, expressed as log-transformed number of counts.
